# When Alexithymia Matters: Distinct Schema–Emotion Processing Profiles of Narcissistic Admiration and Rivalry

**DOI:** 10.3390/brainsci16090990

**Published:** 2026-09-18

**Authors:** Dawid Konrad Ścigała, Matteo Angelo Fabris, Elżbieta Zdankiewicz-Ścigała

**Affiliations:** 1 Department of Psychology, Institute of Psychology, The Maria Grzegorzewska University, Ul. Szczęśliwicka 40, 02-353 Warsaw, Poland; dscigala@aps.edu.pl; 2 Department of Psychology, University of Turin, Via Verdi 10, 10124 Turin, Italy; 3 Faculty of Psychology, SWPS University, Chodakowska 19/31, 03-815 Warsaw, Poland; ezdankiewicz-scigala@swps.edu.pl

**Keywords:** alexithymia, emotion regulation, emotion processing, narcissistic rivalry, narcissistic admiration, early maladaptive schemas, vulnerability–stress model

## Abstract

**Highlights:**

**What are the main findings?**

- Narcissistic Admiration and Rivalry showed distinct early maladaptive schema profiles.
- DIF and EOT added explanatory value for Rivalry, but not for Admiration.

**What are the implications of the main findings?**

- Alexithymia showed a modest, dimension-specific association with threat-reactive narcissistic self-regulation.
- Longitudinal and experimental studies should test the proposed vulnerability–stress process.

**Abstract:**

Background/Objectives: Alexithymia may constrain emotion regulation, but its relevance may differ across personality configurations. This study examined whether narcissistic Admiration and Rivalry are embedded in distinct early maladaptive schema and alexithymia profiles. Methods: A non-clinical adult sample (N = 311) completed the Narcissistic Admiration and Rivalry Questionnaire, the Young Schema Questionnaire–Short Form 3, and the Toronto Alexithymia Scale–20. Analyses examined 18 schemas and three alexithymia components—Difficulty Identifying Feelings (DIF), Difficulty Describing Feelings (DDF), and Externally Oriented Thinking (EOT)—using zero-order correlations, within-domain regressions, hierarchical regressions, and structural equation models. Results: Admiration was associated mainly with Approval/Recognition Seeking and Unrelenting Standards and with lower Social Isolation/Alienation, Defectiveness/Shame, Failure, Subjugation, Emotional Inhibition, and Insufficient Self-Control. Rivalry showed a threat-related profile involving Defectiveness/Shame, Subjugation, Emotional Inhibition, Negativity/Pessimism, and Insufficient Self-Control. Entitlement/Grandiosity was positively associated with both dimensions and did not reliably differentiate them. Alexithymia was essentially unrelated to Admiration and did not improve its schema model. In contrast, adding DIF, DDF, and EOT increased explained variance in Rivalry by 5.5%, a small-to-moderate increment; DIF and EOT made independent contributions, whereas DDF did not. A parsimonious Rivalry structural model reproduced these associations but showed mixed global fit. Conclusions: These cross-sectional findings indicate a modest, dimension-specific contribution of alexithymic processing to Rivalry rather than a general association with narcissistic self-regulation. The EOT findings require particular caution because of the subscale’s limited reliability. Longitudinal and experimental studies are needed to test the proposed vulnerability–stress process.

## 1. Introduction

Narcissistic self-regulation may be shaped both by the cognitive–affective representations through which interpersonal events are interpreted and by the way resulting emotional states are processed and regulated. Early maladaptive schemas and alexithymia provide complementary perspectives on these processes, yet they have rarely been integrated within the same framework. The present study examines whether such integration helps explain the distinct regulatory profiles of narcissistic Admiration and Rivalry.

### 1.1. Narcissistic Admiration and Rivalry as Distinct Regulatory Configurations

Narcissistic self-regulation provides an informative context for examining how differences in emotional processing may become relevant to distinct patterns of personality functioning.

Grandiose narcissistic traits are increasingly conceptualized as multidimensional rather than unitary. Within the Narcissistic Admiration and Rivalry Concept, narcissistic admiration reflects assertive self-enhancement, whereas narcissistic rivalry reflects antagonistic self-protection [1]. Although both dimensions are positively related and serve the broader goal of maintaining a grandiose self, they show markedly different interpersonal and affective correlates.

This distinction is supported by findings on emotion regulation. In a sample of 1018 participants, Cheshure et al. found that narcissistic admiration was negatively associated with problematic responses to emotions and poor recognition of emotions, whereas narcissistic rivalry showed positive associations with both [2]. When admiration and rivalry were entered simultaneously, admiration predicted fewer problematic responses to emotions (β = −0.26) and better emotion recognition (β = −0.31), whereas rivalry predicted more problematic emotional responding (β = 0.41) and poorer emotion recognition (β = 0.26). These findings suggest that admiration and rivalry differ not only in interpersonal strategy but also in their affective-regulatory correlates.

A converging distinction emerges from broader personality models. Rogoza et al. found that Admiration was strongly associated with Extraversion and Intellect/Openness and clustered with the higher-order Plasticity metatrait, whereas Rivalry was associated with low Agreeableness, low Emotional Stability, and impulsivity and mapped onto the opposite pole of Stability [3]. These results position Admiration within an approach- and exploration-oriented configuration and Rivalry within a more unstable and antagonistic regulatory configuration. Krizan and Herlache similarly distinguished approach-dominant boldness from threat-sensitive reactivity as functionally different orientations relevant to narcissistic expression [4]. Developmental correlates show a similarly asymmetric pattern. In a large multi-cohort study of childhood maltreatment and narcissistic dimensions, narcissistic rivalry was positively associated with childhood maltreatment, whereas admiration showed negative associations, with emotional abuse, emotional neglect, and physical neglect contributing most consistently to the differentiation between the two dimensions [5]. This suggests that rivalry may be more strongly embedded in developmental contexts characterized by relational threat and unmet emotional needs, whereas admiration may emerge within a different regulatory architecture.

Taken together, these findings argue against treating admiration and rivalry as neighboring expressions of a single narcissistic process. Rather, they appear to represent distinct regulatory configurations: admiration is more strongly associated with agency, self-enhancement, and comparatively more adaptive emotion-regulation correlates, whereas rivalry is more closely linked to vulnerability, negative affectivity, antagonism, and emotion-regulation difficulties. This distinction raises the question of whether the two narcissistic dimensions are also embedded in different early maladaptive schema and emotion-processing profiles.

### 1.2. Early Maladaptive Schemas as Cognitive–Affective Vulnerability Structures

Early maladaptive schemas (EMS) are enduring cognitive–affective representations concerning the self, others, and interpersonal relationships that are theorized to develop in the context of unmet core emotional needs and adverse relational experiences. Within schema theory, EMS are not conceived as purely cognitive beliefs; they incorporate memories, emotions, cognitions, and bodily sensations, and are reactivated when current experiences are perceived as schema-congruent, often eliciting intense negative affect and maladaptive coping responses. This makes EMS particularly relevant to a vulnerability–stress perspective, because they may provide the relatively stable representational substrate through which interpersonal events acquire affective meaning.

A substantial literature links EMS to insecure attachment. In the first meta-analysis of adult attachment and EMS, Karantzas, Younan, and Pilkington synthesized 15 studies including 3339 participants [6]. Attachment anxiety was positively associated with overall EMS at r = 0.36, attachment avoidance at r = 0.22, and fearful attachment at r = 0.28. The strongest domain-level association for both anxious and avoidant attachment was with Disconnection/Rejection, although the association was significantly larger for attachment anxiety (r = 0.49) than for attachment avoidance (r = 0.31). At the level of specific schemas, attachment anxiety showed particularly strong associations with Abandonment (r = 0.51), Defectiveness/Shame (r = 0.41), Social Isolation/Alienation (r = 0.40), Subjugation (r = 0.44), Approval Seeking (r = 0.40), and Negativity/Pessimism (r = 0.46). Attachment avoidance showed its largest associations with Emotional Deprivation (r = 0.33), Mistrust/Abuse (r = 0.31), Defectiveness/Shame (r = 0.31), Social Isolation/Alienation (r = 0.30), Vulnerability to Harm/Illness (r = 0.29), and Emotional Inhibition (r = 0.39). These findings indicate that attachment insecurity is associated not with a single undifferentiated schema burden but with partially distinct representational profiles.

The distinction is theoretically meaningful. Attachment anxiety is characterized by hyperactivating strategies, heightened negative affect, chronic fears of rejection and abandonment, and a strong need for proximity and approval. Consistent with this profile, the meta-analytic findings showed stronger links with abandonment-related, defectiveness-related, and other-directed schemas. By contrast, attachment avoidance is characterized by deactivating strategies, discomfort with emotional closeness, distrust of others, excessive self-reliance, and suppression of negative affect. Karantzas et al. specifically note that the stronger association between avoidance and Emotional Inhibition is consistent with deactivating strategies that minimize the experience and processing of emotion. Thus, EMS may help specify the cognitive–affective content through which broad attachment orientations become organized into more specific patterns of emotional and interpersonal functioning.

Early maladaptive schemas have also been linked to narcissistic traits, although the pattern varies across narcissistic dimensions. Zeigler-Hill and colleagues showed that different forms of narcissism were associated with different EMS profiles, with Entitlement/Grandiosity emerging as one of the most consistent common correlates across narcissistic forms. At the same time, vulnerable narcissistic features showed broader associations with maladaptive schemas than more grandiose forms. Importantly, the authors also cautioned that some of the strong association involving Entitlement/Grandiosity may reflect overlap in item content between schema and narcissism measures, raising the possibility of criterion contamination. For this reason, Entitlement/Grandiosity is informative as a shared narcissistic marker but should be interpreted cautiously as an etiological or regulatory antecedent.

More recent work using narcissism dimensions closer to the Admiration–Rivalry distinction further supports differential schema profiles. Ray and Fritzon examined Agentic Extraversion and Narcissistic Antagonism in relation to EMS [7]. Agentic Extraversion showed strong positive associations with Entitlement/Grandiosity, Approval/Recognition Seeking, and Unrelenting Standards, whereas Narcissistic Antagonism was more strongly characterized by Mistrust/Abuse, Negativity/Pessimism, Vulnerability to Harm/Illness, and Punitiveness in addition to Entitlement/Grandiosity. The contrast was particularly notable for Mistrust/Abuse, which correlated more strongly with Antagonism than with Agentic Extraversion. This pattern is consistent with the idea that agentic narcissistic functioning is embedded more strongly in status- and recognition-related schemas, whereas antagonistic narcissistic functioning is more strongly embedded in schemas concerning interpersonal threat, distrust, and negative self–other representations.

This distinction is also compatible with the broader NARC literature. Narcissistic admiration is characterized by assertive self-enhancement, status pursuit, and socially expansive self-presentation, whereas rivalry reflects antagonistic self-protection in response to threats to self-worth. If EMS provide enduring representations through which interpersonal events are interpreted, then different EMS may be expected to become particularly relevant to these two narcissistic strategies. Schemas centered on approval, standards, and recognition may be especially compatible with admiration, whereas schemas centered on defectiveness, mistrust, rejection, and threat may be more closely aligned with rivalry.

Taken together, the available evidence suggests that EMS may constitute a cognitive–affective vulnerability layer linking relational history and attachment-related representations with later narcissistic self-regulation. However, existing studies have largely examined EMS in relation either to attachment or to narcissism, rather than testing how specific schemas might connect to different forms of narcissistic functioning through distinct emotional-processing pathways. This unresolved question becomes particularly important when alexithymia is considered not as a unitary deficit, but as a multidimensional pattern of emotion processing.

The unresolved question is therefore not only which schemas characterize different narcissistic dimensions, but whether their affective significance is accompanied by distinct patterns of emotional processing. This issue becomes especially important when alexithymia is considered not as a unitary deficit, but as a multidimensional constraint on the information available for emotion regulation.

### 1.3. Alexithymia as an Emotion-Processing Layer

Alexithymia is best conceptualized here as a multidimensional, trait-like construct rather than a diagnosis or a disorder-specific marker [8]. It involves difficulty identifying feelings (DIF), difficulty describing feelings (DDF), and an externally oriented thinking style (EOT). These components are related but not functionally interchangeable. DIF is most closely linked to undifferentiated negative affect and concurrent distress, DDF concerns the symbolic and interpersonal communication of emotional experience, and EOT reflects reduced attention to and elaboration of internal emotional states. Although alexithymic characteristics show meaningful temporal stability, their measured expression may also vary with psychological distress; the term trait-like therefore does not imply immutability.

A brain–behaviour perspective further supports treating alexithymia as a distributed and transdiagnostic emotion-processing characteristic. Meta-analytic neuroimaging evidence has linked higher alexithymia to altered recruitment of the amygdala, anterior insula, dorsal anterior cingulate, and medial prefrontal regions during emotion processing, although the direction and location of effects vary across task demands and emotional valence [9]. Subjective interoception is also related to alexithymia in a measure- and facet-dependent manner, with stronger associations generally observed for DIF and DDF than for EOT [10]. In autistic participants, empathic insular responses have been associated with alexithymia rather than autism per se [11], while comparative work indicates that alexithymic, autistic, and psychopathic traits relate to distinguishable aspects of social-affective processing [12]. These findings argue against locating any TAS-20 facet in a single brain region or treating EOT as a proxy for a diagnosis or for lack of empathy.

The distinction among alexithymia components is also evident in their relationships with early maladaptive schemas. In a sample of 271 chronic pain patients, Saariaho et al. examined all 18 EMS in relation not only to TAS-20 total scores but also to DIF, DDF, and EOT separately [13]. Several associations were large and clearly differentiated by alexithymia facet. DIF was strongly related to Vulnerability to Harm/Illness (r = 0.512) and also showed substantial associations with Mistrust/Abuse (r = 0.475), Emotional Deprivation (r = 0.488), Defectiveness/Shame (r = 0.488), and Social Isolation/Alienation (r = 0.477). DDF showed particularly strong associations with Mistrust/Abuse (r = 0.517), Emotional Deprivation (r = 0.517), and Emotional Inhibition (r = 0.612). By contrast, EOT showed generally smaller associations with EMS. After controlling for depressive symptoms, several schema–alexithymia associations remained, including DIF with Vulnerability to Harm/Illness (r = 0.322) and DDF with Emotional Inhibition (r = 0.434). These findings suggest that EMS are not uniformly related to global alexithymia, but may be differentially associated with specific forms of emotional-processing difficulty.

This component-specific pattern is consistent with broader evidence linking EMS and alexithymia. Meta-analytic findings indicate that alexithymia is positively associated with the large majority of early maladaptive schemas, with particularly strong associations for Emotional Inhibition and other schemas involving unmet attachment, autonomy, and emotional-expression needs. More recent path-analytic work likewise indicates that EMS are strongly associated with alexithymia and may statistically account for part of the association between childhood adversity and later alexithymic traits [14]. In the study by Zhang et al., EMS and alexithymia correlated at r = 0.653, and EMS fully mediated the cross-sectional association between childhood trauma and alexithymia in the estimated model [15]. Although such findings do not establish temporal causality, they support the view that maladaptive cognitive–affective representations and alexithymic processing are closely interconnected.

The functional significance of alexithymia becomes clearer when considered within contemporary models of emotion regulation. In the process model of emotion regulation, emotions become targets of regulation only after the individual attends to the unfolding emotional response, appraises its meaning for current goals, and decides whether regulation is needed. Subsequent regulation then proceeds through stages of identification, strategy selection, implementation, and monitoring. Preece, Ditzer, and Gross explicitly note that alexithymia involves difficulties attending to and appraising emotional states and may therefore interfere with downstream regulatory decisions across these stages [16]. From this perspective, alexithymia is relevant not merely because emotions are experienced intensely, but because the informational quality of the emotional state available for regulation may be reduced. Empirical evidence supports this interpretation. In a community sample of 501 adults, Preece et al. found that, after controlling for demographic variables and current psychological distress, individuals high in alexithymia reported greater use of expressive suppression, behavioral withdrawal, and ignoring, together with less frequent use of cognitive reappraisal, active problem solving, and seeking social support [17]. The authors interpreted this profile as reflecting emotional avoidance that extends beyond suppression of emotional expression to reduced interpersonal engagement and reduced active coping in response to emotional difficulties.

Importantly, these associations cannot be reduced simply to general psychological distress. Mortezaei et al., using a community sample enriched for psychiatric histories, found that the three TAS-20 components retained significant incremental prediction of emotion-regulation outcomes after controlling for depression, anxiety, and stress [18]. The incremental contribution was largest for global emotion dysregulation and behavioral regulation outcomes. At the facet level, DDF and EOT emerged as the most consistent unique predictors, whereas DIF showed greater attenuation after distress was controlled. The authors therefore concluded that alexithymia captures functionally meaningful variance in emotion regulation that is overlapping with, but not reducible to, concurrent distress.

Taken together, these findings support a view of alexithymia as an emotion-processing layer that may shape how affective activation is translated into regulation. DIF may be especially relevant when negative affect is intense but poorly differentiated, DDF when emotional experience cannot be readily translated into communicable form, and EOT when processing is directed away from internal affective information toward external events and concrete features of the situation. This differentiation is particularly relevant to narcissistic functioning because admiration and rivalry appear to differ markedly in their affective-regulatory correlates. If rivalry is embedded in a threat-related schema configuration characterized by shame, mistrust, rejection sensitivity, and negative affect, then DIF and EOT may become especially relevant to understanding how that affective activation is processed. By contrast, if admiration is embedded more strongly in agency-, recognition-, and status-related schemas, alexithymic processing may play a smaller role.

This possibility provides the basis for a vulnerability–stress account of distinct schema–alexithymia configurations underlying narcissistic admiration and narcissistic rivalry.

### 1.4. A Vulnerability–Stress Account of Schema–Alexithymia–Narcissism Configurations

A vulnerability–stress framework provides a provisional way to organize the expected associations. EMS can be treated as relatively enduring cognitive–affective structures that shape the meaning assigned to interpersonal events, whereas alexithymic characteristics may constrain the identification and elaboration of the accompanying affective state. In the present study, this framework organizes hypotheses about covariance; schema activation, affective arousal, and temporal sequencing were not measured.

The framework is most relevant to Rivalry because antagonistic self-protection is associated with negative affectivity, interpersonal threat, aggression, and vulnerable narcissistic features [1,2,3,4,5]. Schemas centered on defectiveness, mistrust, rejection, or inhibition may therefore co-occur with Rivalry in a threat-sensitive configuration. Within such a configuration, DIF may reflect poor differentiation of activated affect, whereas EOT may reflect reduced attention to internal emotional information. DDF, which more directly concerns communicating an identified state, was expected to contribute less uniquely [16,17,18]. These interpretations describe possible functions of concurrent individual differences rather than an observed process.

A different pattern was expected for Admiration. Its associations with agency, status pursuit, recognition seeking, and approach-oriented functioning suggest that schemas related to achievement and self-enhancement may be relevant without the same degree of threat-related emotional-processing difficulty [1,2,3]. Alexithymia was therefore expected to add little explanatory information after the schema profile of Admiration was represented.

Accordingly, the model distinguishes the content represented by EMS from the quality of emotional information indexed by alexithymia. It predicts that threat-related schemas and DIF/EOT will form a more informative cross-sectional configuration around Rivalry than around Admiration. It does not establish that schemas precede alexithymia or that alexithymia produces Rivalry; reverse and reciprocal processes remain equally compatible with concurrent self-report data.

Longitudinal, experimental, or intensive repeated-measures studies will be required to determine whether schema-congruent interpersonal stress prospectively changes emotional differentiation or internal attention and whether such changes precede antagonistic self-protection. The present analysis is limited to identifying whether the observed pattern is consistent with that future process hypothesis.

### 1.5. The Present Study

The available literature points to an important but insufficiently integrated set of associations. Early maladaptive schemas have been linked to alexithymia, narcissistic traits have been linked to alexithymia, and EMS have also been linked to narcissistic functioning. However, these lines of research have largely developed separately. We are not aware of a study that has simultaneously examined whether specific EMS are differentially associated with distinct alexithymia components and whether these schema–alexithymia configurations distinguish narcissistic admiration from narcissistic rivalry. This gap is particularly relevant because both narcissism and alexithymia are multidimensional constructs, and aggregation may obscure functionally meaningful differences.

The present study therefore examined Admiration and Rivalry as separate narcissistic dimensions, DIF, DDF, and EOT as distinct components of alexithymia, and specific EMS rather than a global schema score. Guided by a vulnerability–stress framework, EMS were conceptualized as relatively stable cognitive–affective vulnerability structures, whereas alexithymic processing was conceptualized as an emotion-processing layer relevant to how schema-related affective activation is identified and elaborated. The aim was not to test a causal sequence, but to determine whether distinct cross-sectional schema–emotion-processing configurations characterize Admiration and Rivalry. Because the YSQ Entitlement/Grandiosity schema overlaps conceptually with narcissistic entitlement and superiority, associations involving this schema were interpreted cautiously because of the possibility of criterion contamination. The primary theoretical focus therefore concerned schema dimensions whose content was not intrinsically narcissistic. Analyses proceeded from a broad mapping of associations among the 18 EMS, DIF, DDF, EOT, Admiration, and Rivalry to increasingly parsimonious regression and structural models testing whether alexithymia added explanatory information beyond schema profiles.

Accordingly, the main hypotheses were:

**H1.**  *Narcissistic Rivalry would be positively associated with EMS reflecting interpersonal threat and negative self-representation, particularly Defectiveness/Shame and Mistrust/Abuse [7,19]*.

**H2.**  *DIF and EOT would explain additional variance in Rivalry beyond the relevant EMS, whereas DDF would show a weaker or non-unique association [2,13,14,15,16,17,18]*.

**H3.**  *Narcissistic Admiration would show a distinct schema profile characterized more strongly by approval-, recognition-, standards-, and self-enhancement-related schemas and by lower levels of schemas indexing social disconnection and emotional inhibition [1,3,7]*.

**H4.**  *DIF, DDF, and EOT would provide little or no incremental explanatory value for Admiration once its schema profile was taken into account [2,3,16,17,18]*.

**H5.**  *Admiration and Rivalry would show distinct schema–emotion-processing configurations, with alexithymic processing contributing selectively to Rivalry rather than functioning as a general correlate of narcissistic self-regulation [1,2,3,4,13,14,15,16,17,18]*.

Because the present data are cross-sectional, these hypotheses concern patterns of association and statistical configuration rather than temporal or causal mechanisms. The vulnerability–stress model serves as a theoretical framework for interpretation, while causal sequencing requires longitudinal, experimental, or intensive repeated-measures designs.

## 2. Materials and Methods

### 2.1. Participants and Procedure

The study was conducted in a non-clinical adult sample recruited through an invitation distributed via the SONA research-participation system. Data were collected online. Participation was voluntary, anonymous, and uncompensated. No dedicated attention-check items were used. After data collection, completion times were reviewed; no implausibly short or unusually long completion times were identified, and no participant was excluded on this basis. The initial dataset included 321 participants. Ten participants were excluded because they lacked complete NARQ data: nine had one or three missing NARQ responses and one had no NARQ responses. No item-level data were missing for the TAS-20 or YSQ-S3. The resulting analytic sample comprised N = 311 participants. It consisted predominantly of women (n = 244, 78.5%), with 61 men (19.6%), two participants identifying as another gender, two preferring not to disclose their gender, and two with missing gender data. Valid four-digit birth-year entries were available for 280 participants; mixed-format or invalid entries were treated as missing. Based on the valid entries, mean age was 28.21 years (SD = 10.27; range = 19–71). Participants completed a battery of self-report questionnaires assessing narcissistic traits, early maladaptive schemas, alexithymia, attachment-related characteristics, and fear of intimacy. The present analyses focused on the NARQ, YSQ-S3, and TAS-20. The study was approved by the The Maria Grzegorzewska University (approval code 0285115). All participants provided informed consent before participation, and the study was conducted in accordance with applicable ethical standards.

### 2.2. Measures

#### 2.2.1. Narcissistic Admiration and Rivalry Questionnaire

Narcissistic admiration and rivalry were assessed using the 18-item Narcissistic Admiration and Rivalry Questionnaire (NARQ; Back et al. [1]). The instrument operationalizes the two higher-order dimensions proposed within the Narcissistic Admiration and Rivalry Concept: Admiration, reflecting assertive self-enhancement, and Rivalry, reflecting antagonistic self-protection. The Polish adaptation by Rogoza, Rogoza, and Wyszyńska [20] confirmed the hierarchical structure of the measure, with six lower-order facets loading on the two higher-order dimensions. Each higher-order dimension is assessed with nine items. In the present study, Admiration and Rivalry were analyzed separately rather than combined into an overall narcissism score. Internal consistency was good for both dimensions, with Cronbach’s α = 0.855 for Admiration and α = 0.869 for Rivalry.

#### 2.2.2. Young Schema Questionnaire–Short Form 3

Early maladaptive schemas were assessed using the Young Schema Questionnaire–Short Form 3 (YSQ-S3) in its Polish adaptation (YSQ-S3-PL) [21]. The questionnaire contains 90 items assessing 18 early maladaptive schemas, with five items representing each schema. The Polish adaptation has been psychometrically evaluated and validated for the assessment of the 18-schema structure. The 18 schemas are: Emotional Deprivation, Abandonment/Instability, Mistrust/Abuse, Social Isolation/Alienation, Defectiveness/Shame, Failure, Dependence/Incompetence, Vulnerability to Harm or Illness, Enmeshment/Undeveloped Self, Subjugation, Self-Sacrifice, Emotional Inhibition, Unrelenting Standards/Hypercriticalness, Entitlement/Grandiosity, Insufficient Self-Control/Self-Discipline, Approval/Recognition Seeking, Negativity/Pessimism, and Punitiveness. Internal consistency across the 18 schema scales ranged from α = 0.61 to α = 0.93. For the present study, scores were calculated for each individual EMS rather than collapsed into broad domain scores. This approach was consistent with the primary aim of identifying schema-specific profiles associated with Admiration and Rivalry and with the three alexithymia components. Entitlement/Grandiosity was retained in the descriptive and comparative analyses but treated cautiously in interpretation because of its conceptual overlap with narcissistic content.

#### 2.2.3. Toronto Alexithymia Scale–20

Alexithymia was assessed using the 20-item Toronto Alexithymia Scale (TAS-20; Bagby et al. [22]) in the Polish-language adaptation by Ścigała et al. [23]. The Polish validation supported the original three-factor structure of the instrument in both non-clinical and alcohol-dependent samples. The TAS-20 comprises three components: Difficulty Identifying Feelings (DIF; 7 items), reflecting difficulty differentiating and recognizing emotional states; Difficulty Describing Feelings (DDF; 5 items), reflecting difficulty verbally communicating emotional experience; and Externally Oriented Thinking (EOT; 8 items), reflecting reduced attention to internal emotional experience and a more externally focused cognitive style. Items are rated on a five-point Likert scale, and five items are reverse-scored. Consistent with the aims of the present study, DIF, DDF, and EOT were analyzed separately rather than using only the TAS-20 total score. In the present sample, internal consistency was α = 0.838 and ω = 0.830 for DIF, α = 0.767 and ω = 0.772 for DDF, and α = 0.620 and ω = 0.625 for EOT. The lower reliability of EOT, which is common in TAS-20 research, limits the precision of facet-specific inferences and was examined in additional item-level and sensitivity analyses.

### 2.3. Statistical Analysis

Statistical analyses were conducted using IBM SPSS Statistics 29 and IBM SPSS Amos 31. Descriptive, correlational, and regression analyses used the complete analytic sample (N = 311). Narcissistic Admiration and Rivalry were treated as separate outcomes, and alexithymia was represented by DIF, DDF, and EOT rather than a single total score. Analyses proceeded from zero-order correlations across all 18 EMS to within-domain regressions, hierarchical regressions, and parsimonious structural models. Within each family of 18 schema comparisons in Table 1 and Table 2, false discovery rate was controlled using the Benjamini–Hochberg procedure. For Table 2, adjustment was applied separately to the Admiration coefficients, Rivalry coefficients, and cross-outcome coefficient-difference tests. The structural models were estimated by maximum likelihood. Indirect effects and cross-outcome differences were evaluated using 5000 bootstrap resamples. Model fit was evaluated with chi-square, CFI, TLI, RMSEA with its 90% confidence interval and close-fit test, and SRMR. No structural path or residual covariance was added solely on the basis of modification indices. The DIF–EOT residual covariance in the Rivalry model was retained because these components share emotion-processing variance not represented by the schema predictors; the more restrictive model without this covariance is reported for comparison. Because the data are cross-sectional, directional paths and indirect effects represent statistical decomposition rather than causal mediation.

Sensitivity analyses addressed sample composition, measurement, influential observations, and precision. Gender was examined through women–men mean comparisons, regression models with gender as a covariate, and women-only analyses; the small number of men precluded reliable multigroup SEM. Univariate extreme scores (|z| > 3.29), multivariate Mahalanobis distance (*p* < 0.001), and Cook’s distance were inspected. Primary regression conclusions were re-evaluated using HC3 heteroskedasticity-consistent standard errors without deleting observations solely because they were flagged by a diagnostic. TAS-20 item functioning was examined in a diagnostic three-correlated-factor CFA, and model-based omega coefficients were calculated. Because item 5 has shown weak EOT loading in the Polish validation, the Rivalry regression was repeated with a seven-item EOT score omitting item 5; the validated eight-item score remained primary. Finally, because the existing dataset was not planned from an a priori power analysis, a post hoc sensitivity analysis—not achieved power—estimated the smallest incremental f^2^ detectable with 80% power at α = 0.05 for the three-predictor alexithymia block.

## 3. Results

### 3.1. Descriptive Statistics and Zero-Order Associations

Table 1 presents descriptive statistics and zero-order correlations for Narcissistic Admiration, Narcissistic Rivalry, the 18 early maladaptive schemas, and the three alexithymia components. The two narcissistic dimensions showed clearly different correlational profiles.

Narcissistic Admiration was essentially unrelated to DIF, DDF, and EOT, whereas Rivalry showed moderate positive associations with all three components (r = 0.415, 0.349, and 0.371, respectively). Thus, the zero-order pattern already indicated that alexithymic characteristics were more relevant to Rivalry than to Admiration.

After Benjamini–Hochberg correction across the 18 EMS correlations for each outcome, Admiration remained positively associated with Entitlement/Grandiosity and Approval/Recognition Seeking and negatively associated with Failure and Emotional Inhibition. The small correlations with Mistrust/Abuse, Social Isolation/Alienation, and Unrelenting Standards/Hypercriticalness did not survive correction. In contrast, all 18 EMS correlations with Rivalry remained significant, with the largest associations involving Defectiveness/Shame, Mistrust/Abuse, Dependence/Incompetence, Vulnerability to Harm/Illness, Subjugation, Emotional Inhibition, Negativity/Pessimism, and Punitiveness.

The EMS correlations with alexithymia were broad but component-specific. All 18 associations with DIF and DDF remained significant after correction. Sixteen of the 18 associations with EOT remained significant; Approval/Recognition Seeking and Unrelenting Standards/Hypercriticalness were the exceptions. DIF was most strongly associated with Negativity/Pessimism and Vulnerability to Harm/Illness, DDF with Emotional Inhibition, and EOT with Enmeshment/Undeveloped Self and Dependence/Incompetence.

Overall, the corrected zero-order results preserved the central contrast: Rivalry showed a broad overlap with threat-related EMS and all alexithymia components, whereas the Admiration profile was narrower and showed negligible overlap with alexithymia.

### 3.2. Schema Profiles of Narcissistic Admiration and Rivalry

To identify schema characteristics that differentiated narcissistic Admiration from Rivalry beyond their zero-order associations, separate multiple regression models were estimated within each of the five YSQ-S3 schema domains (Table 2). All schemas belonging to a given domain were entered simultaneously, and standardized regression coefficients were compared across Admiration and Rivalry. Bootstrap confidence intervals based on 5000 resamples were used to evaluate the stability of differences between the corresponding standardized coefficients.

Within Disconnection/Rejection, Defectiveness/Shame differentiated the outcomes most clearly: it was negatively associated with Admiration (β = −0.267, q = 0.007) and positively associated with Rivalry (β = 0.299, q = 0.002; Δβ = 0.567). Social Isolation/Alienation was independently associated only with lower Admiration, whereas Mistrust/Abuse was associated with both outcomes. Emotional Deprivation was positively associated only with Admiration. All corresponding cross-outcome differences except Mistrust/Abuse survived FDR correction.

Within Impaired Autonomy/Performance, Failure and Enmeshment/Undeveloped Self were independently associated with Admiration. For Rivalry, Vulnerability to Harm/Illness survived correction, whereas Dependence/Incompetence did not (q = 0.066). Only the cross-outcome difference for Failure survived correction. Within Impaired Limits, Entitlement/Grandiosity was positively associated with both outcomes and did not differentiate them, whereas Insufficient Self-Control/Self-Discipline was associated with lower Admiration and higher Rivalry and showed a robust cross-outcome difference.

Within Other-Directedness, Subjugation showed the largest differential association (Δβ = 0.652), predicting lower Admiration and higher Rivalry. Approval/Recognition Seeking was positively associated with both outcomes but more strongly with Admiration. The Self-Sacrifice coefficients were not independently significant, although their cross-outcome difference survived correction. Within Overvigilance/Inhibition, Emotional Inhibition and Negativity/Pessimism were more strongly associated with Rivalry, whereas Unrelenting Standards/Hypercriticalness was associated with Admiration. The small Rivalry coefficient and coefficient difference for Punitiveness did not survive FDR correction (q = 0.072 and 0.085, respectively). Thus, multiplicity correction narrowed several peripheral findings but preserved the principal contrast between the schema profiles of Admiration and Rivalry.

### 3.3. Incremental Contribution of Alexithymia Components

To test whether alexithymia provided explanatory information beyond the schema profiles associated with each narcissistic dimension, hierarchical multiple regression models were estimated separately for Narcissistic Rivalry and Narcissistic Admiration (Table 3). Because the preceding analyses were intended to identify parsimonious schema configurations rather than maximize explained variance by including highly intercorrelated EMS, the Rivalry model included Entitlement/Grandiosity and Defectiveness/Shame, whereas the Admiration model included Entitlement/Grandiosity, Social Isolation/Alienation, and Emotional Inhibition. DIF, DDF, and EOT were entered simultaneously in the second step.

For Narcissistic Rivalry, the schema-only model explained 29.9% of the variance, R^2^ = 0.299. Both Entitlement/Grandiosity (β = 0.304, *p* < 0.001) and Defectiveness/Shame (β = 0.342, *p* < 0.001) made significant unique contributions. Adding DIF, DDF, and EOT in the second step produced a statistically significant but modest 5.5% increase in explained variance, ΔR^2^ = 0.055, ΔF(3, 305) = 8.65, *p* < 0.001, resulting in a final R^2^ = 0.354. In the final model, Entitlement/Grandiosity (β = 0.258, *p* < 0.001) and Defectiveness/Shame (β = 0.227, *p* < 0.001) remained significant. Among the alexithymia components, both DIF (β = 0.184, *p* = 0.006) and EOT (β = 0.173, *p* = 0.002) made independent contributions, whereas DDF did not (β = −0.031, *p* = 0.659). A markedly different pattern emerged for Narcissistic Admiration. The schema-only model, including Entitlement/Grandiosity, Social Isolation/Alienation, and Emotional Inhibition, explained 26.3% of the variance, R^2^ = 0.263. Entitlement/Grandiosity positively predicted Admiration (β = 0.548, *p* < 0.001), whereas Social Isolation/Alienation (β = −0.223, *p* = 0.001) and Emotional Inhibition (β = −0.218, *p* = 0.001) were negatively associated with it. Adding DIF, DDF, and EOT increased explained variance by only 0.7%, ΔR^2^ = 0.007, and did not significantly improve the model, ΔF(3, 304) = 1.00, *p* = 0.395; final R^2^ = 0.271. None of the alexithymia components made a significant unique contribution: DIF (β = 0.103, *p* = 0.152), DDF (β = −0.036, *p* = 0.637), and EOT (β = 0.035, *p* = 0.538). The schema coefficients remained essentially unchanged, with Entitlement/Grandiosity (β = 0.529, *p* < 0.001), Social Isolation/Alienation (β = −0.239, *p* < 0.001), and Emotional Inhibition (β = −0.239, *p* = 0.001) remaining significant. Thus, alexithymia showed a modest and dimension-specific incremental association. DIF and EOT explained additional variance in Rivalry beyond its core schema profile, whereas the entire alexithymia block contributed no meaningful incremental information to Admiration. DDF did not uniquely predict either narcissistic dimension once the remaining variables were taken into account.

### 3.4. Structural Models

**Figure 1:**
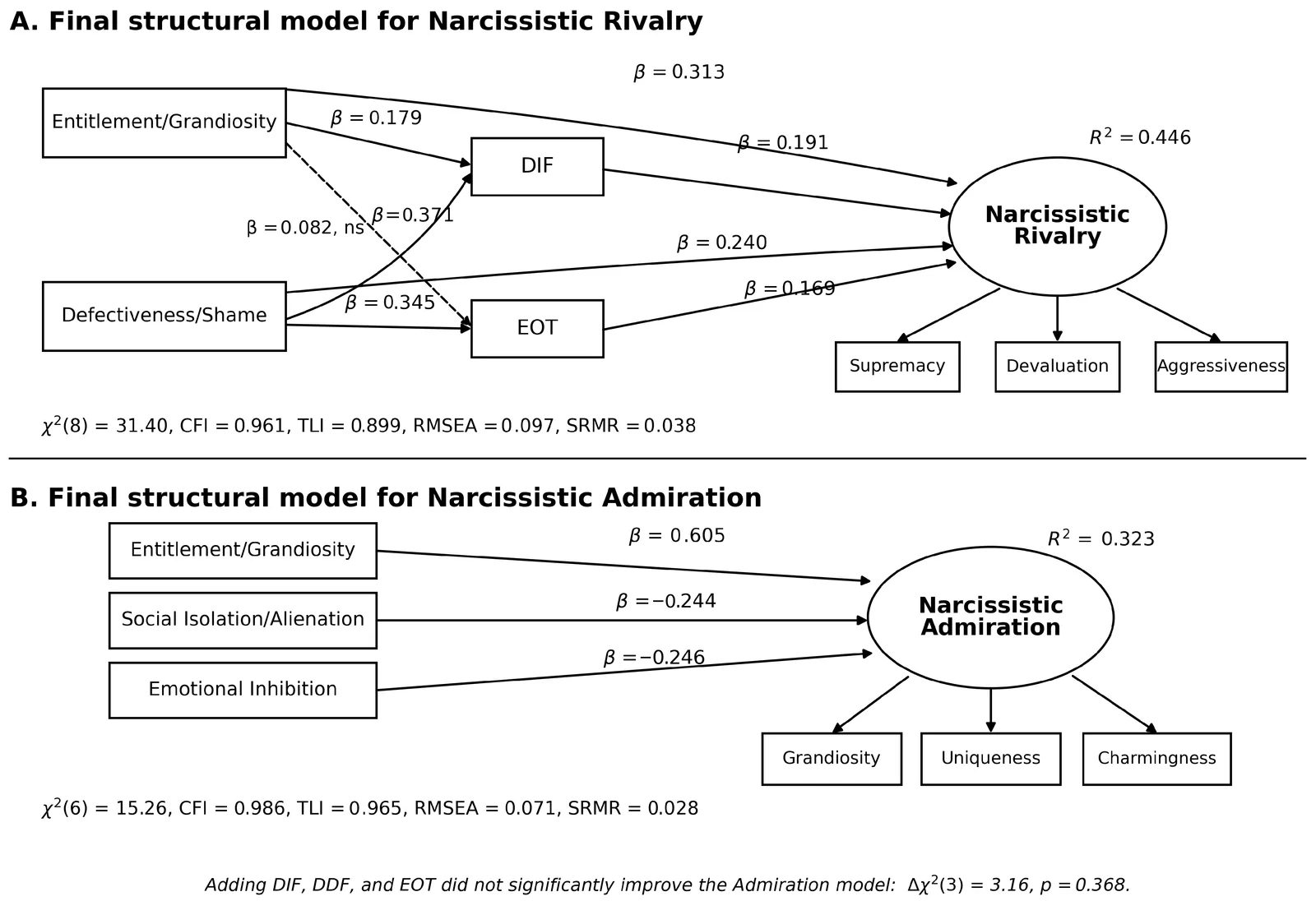
Final structural models for narcissistic Rivalry (Panel (**A**)) and narcissistic Admiration (Panel (**B**)). Standardized path coefficients are shown. Dashed lines indicate nonsignificant paths. Rivalry and Admiration were modeled as latent variables represented by their respective NARQ facets. DIF = Difficulty Identifying Feelings; EOT = Externally Oriented Thinking. Model fit: Rivalry, χ^2^(8) = 31.40, CFI = 0.961, TLI = 0.899, RMSEA = 0.097, 90% CI [0.063, 0.134], pclose = 0.014, SRMR = 0.038; Admiration, χ^2^(6) = 15.26, CFI = 0.986, TLI = 0.965, RMSEA = 0.071, 90% CI [0.027, 0.115], pclose = 0.186, SRMR = 0.028. Adding DIF, DDF, and EOT did not significantly improve the Admiration model, Δχ^2^(3) = 3.16, *p* = 0.368. The residual covariance between DIF and EOT was estimated in the Rivalry model but is omitted from Panel (**A**) for visual clarity.

The final structural models for Narcissistic Rivalry and Narcissistic Admiration are presented in Figure 1.

#### 3.4.1. Narcissistic Rivalry

The final Rivalry model specified Entitlement/Grandiosity and Defectiveness/Shame as schema predictors of DIF and EOT, with direct paths from both schemas and both alexithymia components to latent Narcissistic Rivalry. Rivalry was represented by its three NARQ facets. A residual covariance between DIF and EOT was estimated because the two alexithymia components share variance not accounted for by the schema predictors (Table 4.).

The Rivalry model showed mixed fit, χ^2^(8) = 31.40, CFI = 0.961, TLI = 0.899, RMSEA = 0.097, 90% CI [0.063, 0.134], pclose = 0.014, SRMR = 0.038. CFI and SRMR were favorable, but the elevated RMSEA, significant close-fit test, and marginal TLI preclude characterizing the model as globally well fitting. It should be treated as a parsimonious statistical representation requiring replication. The schema predictors showed clearly differentiated associations with the two alexithymia components. Defectiveness/Shame was positively associated with both DIF (β = 0.371) and EOT (β = 0.345). Entitlement/Grandiosity was associated with DIF (β = 0.179) but showed only a weak association with EOT (β = 0.082). Both schemas also retained direct associations with latent Rivalry: Entitlement/Grandiosity, β = 0.313, and Defectiveness/Shame, β = 0.240. Importantly, both alexithymia components independently contributed to Rivalry, with DIF, β = 0.191, and EOT, β = 0.169. The model explained 22.8% of the variance in DIF, 15.1% of the variance in EOT, and 44.6% of the variance in latent Narcissistic Rivalry. The indirect associations involving Defectiveness/Shame were particularly consistent. Its indirect association with Rivalry through DIF was β = 0.071, 95% CI [0.027, 0.116], and through EOT β = 0.058, 95% CI [0.014, 0.112]. The combined indirect association was β = 0.129, 95% CI [0.068, 0.199]. For Entitlement/Grandiosity, the indirect association through DIF was small but stable, β = 0.034, 95% CI [0.008, 0.069], whereas the EOT pathway was not reliable, β = 0.014, 95% CI [−0.002, 0.041]. Its total indirect association was β = 0.048, 95% CI [0.016, 0.090]. This distinction is substantively important: Defectiveness/Shame was linked to Rivalry both directly and through both forms of alexithymic processing, whereas Entitlement was primarily a direct correlate of Rivalry. A model omitting the residual covariance between DIF and EOT fit the data substantially worse, χ^2^(9) = 47.10, CFI = 0.937, TLI = 0.854, RMSEA = 0.117, SRMR = 0.053, supporting retention of the theoretically plausible residual association between the two alexithymia components.

#### 3.4.2. Narcissistic Admiration

A parallel model was estimated for Admiration. Latent Narcissistic Admiration was represented by its three NARQ facets and regressed on Entitlement/Grandiosity, Social Isolation/Alienation, and Emotional Inhibition. The schema-only model showed good fit, χ^2^(6) = 15.26, CFI = 0.986, TLI = 0.965, RMSEA = 0.071, 90% CI [0.027, 0.115], pclose = 0.186, SRMR = 0.028. Entitlement/Grandiosity was strongly positively associated with Admiration (β = 0.605), whereas Social Isolation/Alienation (β = −0.244) and Emotional Inhibition (β = −0.246) were negatively associated with Admiration. The model explained 32.3% of the variance in latent Admiration. The critical test was whether adding DIF, DDF, and EOT improved this model. To evaluate this formally, the three alexithymia components were included as additional predictors while their covariances with the schema predictors were freely estimated. Allowing the three additional alexithymia paths to Admiration did not significantly improve model fit, Δχ^2^(3) = 3.16, *p* = 0.368. The explained variance increased only from R^2^ = 0.323 to R^2^ = 0.333, an increment of approximately 1%. The structural coefficients for the original schema predictors remained essentially unchanged. Thus, in contrast to Rivalry, alexithymia added no meaningful structural information to Admiration once its schema profile was represented.

### 3.5. Measurement and Robustness Analyses

The diagnostic three-factor TAS-20 CFA showed suboptimal fit, χ^2^(167) = 660.49, CFI = 0.778, TLI = 0.747, RMSEA = 0.098, and SRMR = 0.100. Standardized loadings were generally positive, but item 5 loaded very weakly on EOT (λ = 0.062), consistent with previously reported difficulties involving this item in the Polish adaptation [23]. Model-based reliability was ω = 0.830 for DIF, 0.772 for DDF, and 0.625 for EOT. These findings reinforce the need for caution in interpreting the EOT-specific result rather than supporting a strong claim of measurement precision.

The primary EOT result did not depend on item 5. Omitting that item increased internal consistency from α = 0.620 to 0.676 and omega from 0.625 to 0.656. In the Rivalry regression, the seven-item EOT score remained independently associated with the outcome (β = 0.153, *p* = 0.006), while DIF also remained significant (β = 0.194, *p* = 0.005). The validated eight-item scoring was retained for the primary analyses, and the item-omission model was treated only as a sensitivity check.

Gender sensitivity analyses likewise preserved the central pattern. Among women and men with available binary gender data (n = 305), no mean difference across the focal outcomes, schemas, or alexithymia components survived FDR correction. Gender was unrelated to Rivalry (β = 0.039, *p* = 0.426) and Admiration (β = 0.034, *p* = 0.512) when entered as a regression covariate, and the substantive coefficients changed minimally. In women-only analyses (n = 244), the alexithymia block remained significant for Rivalry (ΔR^2^ = 0.048, *p* = 0.001), with independent DIF (β = 0.210, *p* = 0.013) and EOT (β = 0.172, *p* = 0.007) coefficients, but remained nonsignificant for Admiration (ΔR^2^ = 0.008, *p* = 0.487). Women-only SEM estimates were similar in direction and magnitude to the full-sample estimates.

Outlier diagnostics identified three cases exceeding the multivariate Mahalanobis criterion (*p* < 0.001), but no observation was removed solely on that basis. HC3 robust inference preserved the Rivalry associations for DIF (*p* = 0.013) and EOT (*p* = 0.002) and the null alexithymia coefficients for Admiration. Finally, sensitivity analysis indicated that N = 311 provided 80% power at α = 0.05 to detect an incremental effect of approximately f^2^ = 0.036 for the three-variable alexithymia block. The observed incremental effect was f^2^ = 0.085 for Rivalry and f^2^ = 0.010 for Admiration. Because the analysis used an existing dataset, these values describe detectable effect size and observed magnitude rather than an a priori design calculation or achieved power.

## 4. Discussion

### 4.1. Distinct Schema Architectures of Admiration and Rivalry

The present findings indicate that narcissistic Admiration and Rivalry are embedded in markedly different early maladaptive schema configurations. This distinction was evident both at the zero-order level and in within-domain regression models. Admiration was characterized primarily by higher Approval/Recognition Seeking and Unrelenting Standards/Hypercriticalness, together with comparatively lower Defectiveness/Shame, Social Isolation/Alienation, Failure, Subjugation, Emotional Inhibition, and Insufficient Self-Control/Self-Discipline. Rivalry showed the opposite pattern, with especially strong differentiation involving Defectiveness/Shame, Subjugation, Emotional Inhibition, Negativity/Pessimism, and Insufficient Self-Control/Self-Discipline. Thus, the distinction between the two narcissistic dimensions was not simply one of greater versus lesser schema burden, but of qualitatively different schema content. This pattern is highly consistent with prior work portraying Admiration and Rivalry as functionally distinct narcissistic strategies [1,3]. In the NARC framework, Admiration reflects assertive self-enhancement, whereas Rivalry reflects antagonistic self-protection [1]. Rogoza et al. showed that Admiration was embedded in a Plasticity-like personality configuration, whereas Rivalry mapped onto the opposite pole of Stability, Alpha-minus [3]. Admiration was strongly associated with Extraversion and Intellect, whereas Rivalry was associated with low Agreeableness, low Emotional Stability, and impulsivity. In their regression models, Extraversion predicted Admiration at β = 0.60 and Rivalry at β = −0.21, whereas Emotional Stability predicted Admiration at β = 0.35 and Rivalry at β = −0.38; Agreeableness showed a similar contrast, β = 0.21 versus β = −0.40 [3]. Their factor-analytic results likewise placed Admiration within Plasticity and Rivalry within a Stability-related configuration [3], reinforcing the view that the two dimensions reflect different broader modes of functioning rather than simply different levels of one underlying narcissistic tendency. The schema profile observed here closely parallels this distinction. Approval/Recognition Seeking and Unrelenting Standards are compatible with a self-enhancing, achievement- and status-oriented configuration in which external validation, distinction, and high personal standards may support the pursuit of admiration. In contrast, Defectiveness/Shame, Social Isolation, Subjugation, Emotional Inhibition, and Negativity/Pessimism describe a markedly different representational field—one characterized by negative self-evaluation, anticipated rejection, constrained emotional expression, and a more threat-saturated view of interpersonal experience. This pattern is broadly consistent with the interpretation of Rivalry as a self-protective strategy organized around interpersonal antagonism rather than expansive self-promotion [1]. The Narcissism Spectrum Model provides a useful complementary framework for understanding this divergence [4]. Krizan and Herlache proposed that narcissistic expressions share a common core of entitlement and self-importance but differ in their underlying functional orientations. Grandiose expressions are linked to boldness and approach, whereas vulnerable expressions are linked to reactivity and avoidance [4]. Although Rivalry should not be equated with vulnerable narcissism, the present Rivalry profile appears more compatible with a reactivity-like configuration than the Admiration profile. Reactivity is described as a threat-focused orientation characterized by vigilance, emotional dysregulation, negative emotionality, anger, and avoidance tendencies [4]. This closely resembles the schema constellation identified here around Defectiveness/Shame, Negativity/Pessimism, Emotional Inhibition, and Subjugation. At the same time, the present findings caution against collapsing Rivalry into narcissistic vulnerability. Krizan and Herlache explicitly characterize the NARQ Rivalry subscale as primarily tapping entitlement and antagonism, with strong links to manipulativeness and conflict, rather than as a direct measure of vulnerable narcissism [4]. The current results are therefore better interpreted as showing that Rivalry is embedded in a more threat-sensitive and negatively valenced schema configuration, not that it is equivalent to vulnerability. This distinction is important because it preserves the conceptual specificity of the NARC model while allowing its antagonistic component to be situated within a broader affective-regulatory context.

One of the most informative findings was that several schemas showed opposite-sign associations with Admiration and Rivalry. Defectiveness/Shame, Social Isolation/Alienation, Subjugation, Emotional Inhibition, and Insufficient Self-Control/Self-Discipline were associated with lower Admiration but higher Rivalry in the multivariable models. These cross-over effects suggest that the two dimensions may be organized around partly opposing regulatory configurations. Admiration appears more compatible with outward engagement, standards, recognition seeking, and lower self-representational threat, whereas Rivalry appears more compatible with self-protective responses emerging in a context of shame, alienation, inhibition, and negative expectations. This contrast is also consistent with the broader literature on self-esteem and personality correlates. Rogoza et al. found Admiration to be positively associated with self-esteem and Rivalry negatively associated with it, while Rivalry—but not Admiration—was positively linked to impulsivity [3]. Such findings are compatible with the possibility that Admiration operates more often within an approach-oriented system in which self-enhancement can be pursued directly, whereas Rivalry becomes particularly relevant when self-worth is challenged or threatened [1,3]. Taken together, the present schema findings extend prior personality-level evidence by showing that the distinction between Admiration and Rivalry is also visible at the level of early maladaptive representations. The two dimensions appear to share some narcissistic content while being embedded in different cognitive–affective and regulatory environments. This divergence provides the necessary background for understanding the central finding of the present study: alexithymia was incrementally relevant to Rivalry, but not to Admiration.

### 4.2. When Alexithymia Matters: Emotion Processing in Rivalry

A notable finding was that alexithymia contributed incrementally to narcissistic Rivalry after the core schema profile was represented. The 5.5% increase in explained variance was statistically reliable and corresponds to a small-to-moderate incremental effect; it should not be interpreted as a dominant explanation of Rivalry. DIF and EOT made independent contributions, whereas DDF did not. In the structural model, both components retained paths to latent Rivalry, and Defectiveness/Shame showed indirect statistical associations through DIF and EOT. Because the model fit was mixed and all variables were concurrent, these paths summarize covariance rather than demonstrate a mechanism.

The DIF–EOT distinction is theoretically plausible but should be regarded as provisional. DIF concerns difficulty identifying and differentiating internal affective states, whereas EOT concerns reduced attention to internal emotional experience; DDF more directly concerns communicating emotional states [8,17,18]. However, EOT had limited internal consistency and weak item-level structure in this sample. Its unique regression coefficient was robust to omitting item 5, but the evidence supports cautious replication rather than a strong facet-specific conclusion.

This interpretation is consistent with contemporary models of alexithymia and emotion regulation. Preece et al. conceptualize alexithymia as involving difficulties attending to and appraising emotional states, processes that are necessary for effective emotion regulation [16,17]. Within the process model of emotion regulation, poor-quality information about one’s emotional state can impair decisions at the identification, selection, implementation, and monitoring stages [16]. In a community sample of 501 adults, higher alexithymia was associated with greater expressive suppression, withdrawal, and ignoring, and with lower use of cognitive reappraisal, active problem solving, and social support, even after controlling for psychological distress [17].

The wider neuroscience literature provides context but does not justify a one-to-one neural interpretation of the present self-report associations. Alexithymia has been associated with distributed differences in salience, interoceptive, and emotion-processing systems [9,10,11]. At the same time, work comparing alexithymic, autistic, and psychopathic traits shows that superficially similar social-affective difficulties may arise from distinguishable processes [11,12,24]. Blair’s model of psychopathy emphasizes impaired processing of others’ distress and atypical amygdala–ventromedial prefrontal function [24], constructs not assessed here. EOT therefore cannot be interpreted from the present data as blame externalization, lack of empathy, or reduced insight; such interpretations would require direct measures of those processes.

The present findings extend this literature by suggesting that alexithymic processing may become especially relevant within a threat-sensitive narcissistic configuration. Rivalry was associated with schemas such as Defectiveness/Shame, Emotional Inhibition, Negativity/Pessimism, Subjugation, and Social Isolation/Alienation, all of which point toward a representational field characterized by negative self-evaluation, interpersonal threat, and constrained emotional engagement. Within such a context, DIF may reflect difficulty transforming heightened affective activation into differentiated emotional knowledge, whereas EOT may reflect reduced internal elaboration of that activation. In functional terms, the emotional state may be strongly experienced while remaining insufficiently represented as specific, internally processed emotional information.

This distinction is especially relevant to Rivalry because antagonistic self-protection may depend on how self-relevant threat is processed. The Narcissism Spectrum Model describes reactive narcissistic functioning as organized around vigilance for threat, negative emotionality, frustration, anger, and avoidance-oriented regulation [4]. Although Rivalry is not equivalent to narcissistic vulnerability, its schema profile and emotion-processing correlates are compatible with a more reactive and threat-sensitive mode of functioning. In this sense, alexithymia may not represent a general deficit associated with narcissism, but rather a processing style whose functional significance increases under conditions of self-relevant threat.

The specific role of DIF is consistent with recent evidence that this component is especially closely tied to undifferentiated negative affect and current distress. Mortezaei et al. found that DIF showed the strongest overlap with depression, anxiety, and stress, although it still contributed uniquely to some emotion-regulation outcomes after distress was controlled. By contrast, EOT was substantially less related to distress and retained distinctive associations with cognitive and behavioral regulation [18]. This divergence is useful for interpreting the present model: DIF may index poor differentiation of activated negative affect, whereas EOT may index reduced introspective processing of that affect once activated.

The fact that DDF did not retain a unique association with Rivalry is equally informative. DDF appears to be more closely tied to interpersonal communication of emotion than to the internal recognition or elaboration of affective states [17,18]. Its lack of unique contribution in the present model suggests that the emotion-processing configuration associated with Rivalry may be centered primarily on internal access and differentiation rather than on verbal communication per se. This interpretation is also consistent with Saariaho et al., who found particularly strong associations of DDF with Emotional Inhibition and other relationally oriented EMS [13].

Taken together, these results suggest a functional rather than purely deficit-based interpretation of alexithymia. In the context of Rivalry, alexithymic processing may operate as a mode of reduced access to the meaning of affective activation, limiting the extent to which threat-related emotional states are differentiated and internally elaborated. This may reduce the flexibility of subsequent emotion-regulation responses and increase the relative accessibility of externally directed, antagonistic forms of self-protection. Importantly, this interpretation is consistent with, but not established by, the present cross-sectional data. Future experimental and intensive longitudinal research is needed to test whether schema-congruent interpersonal threat prospectively increases affective activation and whether DIF and EOT shape the transition from that activation to Rivalry-related responses.

### 4.3. Why Alexithymia Does Not Matter for Admiration

In contrast to Rivalry, alexithymia contributed little to narcissistic Admiration. At the zero-order level, Admiration was essentially unrelated to DIF, DDF, and EOT, and the addition of all three alexithymia components to the schema model increased explained variance by only approximately 1%. None of the alexithymia components showed a unique association with Admiration, and the original schema paths remained virtually unchanged. This pattern suggests that alexithymic processing is not a general feature of narcissistic functioning, but becomes relevant only within particular affective-regulatory configurations.

A useful framework for understanding this null pattern is provided by the concept of Plasticity. Rogoza et al. showed that Admiration clusters with Extraversion and Intellect/Openness and corresponds closely to the higher-order Plasticity metatrait [3]. In their factor-analytic model, Admiration loaded within the same broader configuration as Extraversion and Intellect, whereas Rivalry was embedded in the opposite pole of Stability [3]. At the level of specific traits, Admiration was strongly associated with Extraversion and Intellect, whereas Rivalry was characterized instead by low Agreeableness, low Emotional Stability, and greater impulsivity [3].

Plasticity is particularly relevant because it reflects behavioral and cognitive exploration, responsiveness to novelty, and the capacity to generate new goals and strategies [3]. Rogoza et al. explicitly interpret the Admiration profile in these terms, linking it to exploration, openness to change, and adaptation to novelty [3]. This is consistent with the present schema findings. Approval/Recognition Seeking and Unrelenting Standards/Hypercriticalness appear compatible with an outwardly directed, achievement-oriented mode of self-regulation in which the individual actively pursues recognition, distinction, and self-enhancement rather than primarily defending against perceived interpersonal threat.

The Narcissism Spectrum Model provides a converging account. Krizan and Herlache describe grandiose narcissistic expression as grounded in boldness, an approach-dominant orientation characterized by heightened reward sensitivity, extraversion, assertiveness, positive affect, and active pursuit of self-aggrandizing goals [4]. They further emphasize that grandiose self-regulation is organized around the pursuit of status, admiration, power, and social reward through self-enhancing strategies [4].

From this perspective, the absence of alexithymia effects in Admiration is theoretically meaningful. If Admiration is embedded in a predominantly approach-oriented, exploratory, and reward-seeking system, the central regulatory task may be to maximize opportunities for self-enhancement rather than to manage highly aversive internal activation. In such a configuration, the quality of emotion identification or the degree of externally oriented emotional processing may simply be less consequential for maintaining the narcissistic strategy.

This interpretation does not imply that Admiration is emotionally neutral or characterized by low arousal. On the contrary, Plasticity and boldness are compatible with substantial activation, but the activation is more likely to be linked to approach, reward anticipation, novelty, dominance, and social opportunity than to negatively valenced threat [3,4]. Krizan and Herlache note that grandiosity is associated with higher positive affect and strong behavioral activation, while its links with distress and negative affect are weaker [4]. Thus, the key distinction may concern not the presence versus absence of arousal, but the functional meaning and valence of the activation.

This helps clarify why alexithymic processing may add little to Admiration. DIF is particularly relevant when affective activation is difficult to identify and differentiate, whereas EOT may become important when internal emotional information is minimized or insufficiently elaborated [16,17,18]. In an approach-dominant configuration, however, self-regulatory behavior may proceed efficiently through external goal pursuit, social engagement, novelty seeking, and self-promotion without requiring the same degree of threat-related internal emotion processing. The system may therefore remain behaviorally organized even when emotional self-reflection is not central to the regulatory task.

The present schema pattern is consistent with this interpretation. Admiration was associated with higher Approval/Recognition Seeking and Unrelenting Standards and with lower Social Isolation/Alienation and Emotional Inhibition. This constellation is more consistent with active engagement in the social environment than with withdrawal, affective constriction, or threat-focused processing. In this sense, the absence of alexithymia effects is not simply a statistical null, but an informative indication that Admiration may rely on a different regulatory architecture.

The distinction also fits Rogoza et al.’s broader conclusion that Admiration represents the more Plastic, exploratory side of narcissism, whereas Rivalry is associated with Instability and antagonistic control of the environment [3]. Their findings showed that separating Admiration and Rivalry reveals opposite relations with broader personality functioning that disappear when narcissism is treated globally [3]. The present study extends this differentiation by suggesting that the same asymmetry applies to alexithymic emotion processing: it is relevant to Rivalry but not to Admiration.

Taken together, these findings suggest that alexithymia matters when narcissistic self-regulation is embedded in a threat-reactive affective context, but not necessarily when it is organized around Plasticity, approach, and self-enhancement. Admiration may therefore represent a narcissistic strategy in which behavioral and cognitive exploration support flexible goal-directed regulation, reducing the functional relevance of alexithymic processing for the pursuit of status and recognition.

### 4.4. Entitlement as a Shared Core Rather than a Differentiating Mechanism

Entitlement/Grandiosity occupied a distinctive position in the present findings. Unlike most other schemas, it was positively associated with both narcissistic Admiration and Rivalry and did not reliably differentiate between them. In the within-domain models, Entitlement/Grandiosity was a strong predictor of both Admiration and Rivalry, while the difference between the corresponding standardized coefficients was not significant. This pattern suggests that entitlement-related content may represent a shared narcissistic feature rather than a mechanism that explains why Admiration and Rivalry diverge.

This interpretation is closely aligned with the Narcissism Spectrum Model. Krizan and Herlache place entitled self-importance at the center of the narcissism spectrum and argue that different narcissistic expressions share this common phenotypic core while differing in their broader functional orientations [4]. In their formulation, entitlement and self-importance provide the common core of narcissistic personality, whereas boldness and reactivity help shape how this core is expressed [4]. This distinction maps well onto the present findings: Entitlement/Grandiosity was common to both Admiration and Rivalry, but the surrounding schema and emotion-processing architecture differed markedly.

The same point is visible in the NARC framework. Admiration and Rivalry are conceptualized as distinct strategies serving the broader narcissistic goal of maintaining a grandiose self [1]. Admiration pursues this goal through assertive self-enhancement, whereas Rivalry does so through antagonistic self-protection [1]. From this perspective, entitlement may represent a shared motivational or self-representational background against which the two strategies differentiate, rather than the source of that differentiation itself.

The present results therefore support a hierarchical interpretation. At one level, Entitlement/Grandiosity may index a common narcissistic substrate. At another level, the specific configuration in which this substrate is embedded appears to differentiate the two narcissistic dimensions. For Admiration, entitlement co-occurred with higher Approval/Recognition Seeking and Unrelenting Standards and with lower Social Isolation and Emotional Inhibition. For Rivalry, entitlement was embedded in a profile involving Defectiveness/Shame, threat-related schemas, DIF, and EOT. The same shared narcissistic content may therefore be organized within different regulatory contexts.

At the same time, the interpretation of YSQ Entitlement/Grandiosity requires caution because of its conceptual overlap with narcissistic entitlement. Zeigler-Hill et al. noted that associations between narcissism and the Entitlement schema may be inflated by overlapping construct or item content, because narcissism measures themselves often contain entitlement-related material [19]. This raises the possibility of criterion contamination and makes it difficult to interpret YSQ Entitlement/Grandiosity straightforwardly as a developmental antecedent or causal vulnerability.

For this reason, the present findings are more informative where the model extends beyond Entitlement/Grandiosity. The fact that Rivalry remained linked to Defectiveness/Shame, DIF, and EOT, whereas Admiration showed a different pattern involving Social Isolation and Emotional Inhibition, suggests that the differentiation between the two narcissistic dimensions cannot be reduced to shared entitlement content. Rather, entitlement may serve as a common structural marker, while the theoretically meaningful differences lie in the surrounding schema and emotion-processing configuration.

This interpretation is also consistent with Krizan and Herlache’s broader warning against tautological theorizing in narcissism research. They argue that progress requires linking narcissistic constructs to more distal psychological processes rather than merely predicting narcissistic behavior with measures that already contain overlapping narcissistic content [4]. The present study follows this logic by treating entitlement as a shared marker and focusing interpretively on the broader representational and emotion-processing patterns that distinguish Admiration from Rivalry.

Taken together, these findings suggest that entitlement may define what Admiration and Rivalry share, whereas schema content and emotion-processing dynamics help explain how they differ.

### 4.5. Implications for a Vulnerability–Stress Account of Narcissistic Regulation

The findings are consistent with a limited vulnerability–stress interpretation in which Admiration and Rivalry are situated in different cognitive–affective contexts around partly shared entitlement-related content. Admiration was associated with a more approach-oriented profile, whereas Rivalry was embedded in a more threat-related schema configuration to which DIF and EOT added modest explanatory information. This pattern accords with dimensional models that distinguish approach-oriented boldness from threat-reactive narcissistic functioning [3,4].

At the level measured here, EMS describe recurring meanings assigned to the self and interpersonal events, while alexithymia describes aspects of access to emotional information. Their concurrent configuration around Rivalry is compatible with the possibility that threat-related representations become especially consequential when affect is difficult to identify or receives limited internal attention [16,17,18]. The data do not show that a schema was activated, that alexithymia followed that activation, or that either process generated antagonistic behavior.

This qualification also limits the interpretation of the directional SEM arrows. Reversing the structural direction would not provide an independent test because models based on the same cross-sectional covariance matrix can be statistically equivalent. We therefore treat the estimated paths and indirect effects as one theoretically organized decomposition of association, not evidence that the proposed temporal ordering is correct.

The framework is nonetheless useful for generating testable process hypotheses. Ecological momentary assessment could examine whether schema-relevant interpersonal events precede changes in affective arousal, emotion clarity, externally oriented processing, and state Rivalry. Experimental work could combine self-relevant threat manipulations with subjective, behavioral, and psychophysiological measures. Such designs could distinguish mediation from moderation and establish whether the proposed dynamics are specific to Rivalry rather than Admiration.

Thus, the present contribution is narrower than a demonstration of a vulnerability–stress mechanism. It identifies a replicable candidate configuration: shared entitlement-related content, different schema profiles for Admiration and Rivalry, and a modest incremental association of DIF and EOT with Rivalry. The temporal and neural processes that might produce this configuration remain open questions.

## 5. Limitations and Future Directions

Several limitations should be considered when interpreting the present findings. First, the study was cross-sectional. Although the proposed vulnerability–stress framework specifies a temporal sequence in which schema-congruent threat may be followed by affective activation, alexithymic processing, and narcissistic self-regulation, the present data cannot establish this sequence. EMS, alexithymia, and narcissistic traits were assessed concurrently, and the structural models therefore represent patterns of association rather than causal mechanisms.

Second, all constructs were assessed using self-report measures. This is particularly relevant for alexithymia, because difficulties in identifying and describing emotional states may themselves limit the accuracy of self-reported emotional functioning. Future studies should therefore incorporate multimethod assessment, including structured interviews, behavioral tasks, informant reports, and physiological indices of emotional activation.

Third, the study did not include a direct measure of momentary negative affect or general psychological distress. This is important because DIF tends to overlap more strongly with current depression, anxiety, and stress, whereas EOT appears to retain more distinctive associations with emotion-regulation outcomes after distress is controlled [18]. The present data therefore cannot determine the extent to which concurrent negative affect contributed to the observed Rivalry–DIF associations. Future studies should include explicit measures of affective distress alongside alexithymia.

Fourth, the proposed vulnerability–stress account assumes that threat-related schemas become dynamically activated in specific interpersonal contexts, yet neither momentary schema activation nor affective arousal was directly measured. This limits the extent to which the present findings can distinguish between stable trait covariance and context-dependent regulatory processes. Ecological momentary assessment and intensive longitudinal designs would be especially valuable for testing whether schema-relevant interpersonal events prospectively predict changes in affective arousal, emotion clarity, externally oriented processing, and state-like expressions of Rivalry.

Fifth, recruitment through the SONA system and online self-selection may limit representativeness. Individuals willing and able to respond to a research-participation invitation may differ systematically from the wider adult population, and the uncompensated format may have further shaped participation. Although post-collection review identified no implausibly short or unusually long completion times, no dedicated attention-check items were administered; subtler inattentive responding therefore cannot be ruled out. Replication using probability-based or more heterogeneous recruitment channels and explicit response-quality checks is warranted.

Sixth, the sample was predominantly female. Although gender-adjusted analyses and women-only sensitivity analyses indicated that the core pattern was robust, the relatively small number of men precluded reliable multigroup structural modeling. Replication in more gender-balanced samples is therefore needed.

Seventh, Entitlement/Grandiosity requires cautious interpretation because of conceptual overlap with narcissistic entitlement. Its strong association with both Admiration and Rivalry may partly reflect criterion contamination rather than a distinct etiological or regulatory process [19]. Future studies should examine whether the present architecture replicates when entitlement is operationalized with measures that are less content-overlapping with narcissistic outcomes.

Eighth, the TAS-20 EOT subscale showed limited internal consistency (α = 0.620; ω = 0.625), and the standard three-factor item model showed suboptimal fit. Item 5 had a particularly weak loading, consistent with previous findings for the Polish adaptation [23]. Although the EOT coefficient remained significant when item 5 was omitted, this sensitivity result does not eliminate the broader measurement limitation. The EOT-specific interpretation should therefore be considered provisional and replicated using alternative alexithymia instruments with stronger assessment of externally oriented emotional processing.

A particularly important direction for future research is to test the proposed functional role of alexithymia directly. Experimental paradigms could manipulate self-relevant interpersonal threat and assess whether individuals high in threat-related EMS show greater physiological activation, poorer emotion differentiation, and greater externally oriented processing, followed by stronger antagonistic responses. Such work could determine whether DIF and EOT mediate, moderate, or otherwise shape the transition from schema activation to Rivalry-related behavior.

Longitudinal research is equally important for clarifying developmental pathways. The present findings are compatible with a model in which early relational experiences contribute to enduring schema vulnerability, which in turn becomes linked to specific patterns of emotional processing and narcissistic self-regulation. However, equifinality and multifinality should be expected: similar schema profiles may lead to different outcomes depending on temperament and context, while similar narcissistic expressions may arise through different developmental routes [4].

Finally, future studies should examine whether the present distinction generalizes beyond NARQ Admiration and Rivalry. In particular, it would be useful to test whether the same schema–emotion-processing architecture differentiates grandiose, vulnerable, and antagonistic narcissistic dimensions assessed with other instruments, and whether alexithymia remains specifically relevant to threat-reactive rather than approach-oriented narcissistic functioning.

## 6. Conclusions

The present cross-sectional study identified different schema–emotion-processing configurations around narcissistic Admiration and Rivalry. Admiration was associated with a more approach-oriented schema profile, and alexithymia added little explanatory information. Rivalry was associated with a broader threat-related schema profile, and DIF and EOT provided a statistically reliable but modest increment beyond Entitlement/Grandiosity and Defectiveness/Shame.

Entitlement/Grandiosity was associated with both outcomes and did not distinguish them reliably. Given its content overlap with narcissistic characteristics, it is best treated as a shared correlate rather than a developmental or causal mechanism. The more informative contrast lies in the surrounding schema and alexithymia associations.

The findings therefore suggest that alexithymia is not uniformly associated with narcissistic self-regulation. Its contribution was specific to the Rivalry model in this sample, but the modest effect size, mixed SEM fit, cross-sectional design, and limited EOT reliability constrain mechanistic and facet-specific conclusions. The vulnerability–stress account remains a hypothesis for longitudinal, experimental, and intensive repeated-measures research rather than a process demonstrated by the present data.

## Figures and Tables

**Table 1:** Descriptive Statistics, Internal Consistency, and Zero-Order Correlations of Early Maladaptive Schemas with Narcissistic Admiration, Narcissistic Rivalry, and Alexithymia Components.

Variable	M	SD	α	Admiration	Rivalry	DIF	DDF	EOT
Emotional Deprivation	2.29	1.17	0.847	0.083	0.373 ***	0.375 ***	0.401 ***	0.369 ***
Abandonment/Instability	3.02	1.27	0.845	−0.006	0.331 ***	0.450 ***	0.277 ***	0.184 **
Mistrust/Abuse	2.78	1.19	0.848	0.117	0.454 ***	0.432 ***	0.330 ***	0.229 ***
Social Isolation/Alienation	2.84	1.25	0.872	−0.128	0.408 ***	0.424 ***	0.397 ***	0.261 ***
Defectiveness/Shame	2.12	1.25	0.927	−0.056	0.473 ***	0.449 ***	0.454 ***	0.381 ***
Failure	2.53	1.29	0.886	−0.164 *	0.389 ***	0.439 ***	0.342 ***	0.300 ***
Dependence/Incompetence	2.40	1.07	0.800	−0.067	0.451 ***	0.449 ***	0.374 ***	0.400 ***
Vulnerability to Harm/Illness	2.65	1.25	0.838	−0.006	0.425 ***	0.512 ***	0.305 ***	0.248 ***
Enmeshment/Undeveloped Self	2.14	1.11	0.856	0.083	0.393 ***	0.374 ***	0.331 ***	0.426 ***
Subjugation	2.55	1.09	0.777	−0.043	0.415 ***	0.480 ***	0.404 ***	0.307 ***
Self-Sacrifice	3.26	1.00	0.741	0.105	0.162 **	0.265 ***	0.164 **	0.118 *
Emotional Inhibition	2.55	1.08	0.799	−0.153 *	0.410 ***	0.438 ***	0.520 ***	0.369 ***
Unrelenting Standards/Hypercriticalness	3.36	1.12	0.783	0.136	0.278 ***	0.415 ***	0.240 ***	0.027
Entitlement/Grandiosity	2.85	0.87	0.606	0.368 ***	0.452 ***	0.340 ***	0.245 ***	0.232 ***
Insufficient Self-Control/Self-Discipline	3.14	1.10	0.800	0.020	0.361 ***	0.386 ***	0.298 ***	0.208 ***
Approval/Recognition Seeking	3.50	1.09	0.796	0.319 ***	0.366 ***	0.332 ***	0.176 **	0.098
Negativity/Pessimism	3.09	1.22	0.837	−0.040	0.404 ***	0.527 ***	0.327 ***	0.123 *
Punitiveness	2.52	1.07	0.841	−0.056	0.398 ***	0.463 ***	0.415 ***	0.285 ***
Narcissistic Admiration	3.34	0.90	0.855	—	0.199 ***	0.064	−0.036	0.027
Narcissistic Rivalry	2.42	0.93	0.869	0.199 ***	—	0.415 ***	0.349 ***	0.371 ***
DIF	18.40	6.37	0.838	0.064	0.415 ***	—	0.703 ***	0.363 ***
DDF	12.61	4.31	0.767	−0.036	0.349 ***	0.703 ***	—	0.487 ***
EOT	17.11	4.50	0.620	0.027	0.371 ***	0.363 ***	0.487 ***	—

Note. N = 311. NARQ and YSQ-S3 values are mean item scores; TAS-20 facet values are summed scores. DIF = Difficulty Identifying Feelings; DDF = Difficulty Describing Feelings; EOT = Externally Oriented Thinking. For the 18 EMS rows, asterisks indicate Benjamini–Hochberg-adjusted significance within each outcome family: * q < 0.05, ** q < 0.01, *** q < 0.001. Correlations among the focal outcome variables are shown with unadjusted significance levels.

**Table 2:** Within-Domain Regression Models Predicting Narcissistic Admiration and Rivalry from Early Maladaptive Schemas.

EMS Domain/Schema	Admiration β	Adm. qBH	Rivalry β	Riv. qBH	Δβ	95% Bootstrap CI
Disconnection/Rejection						
Emotional Deprivation	0.258	0.004	−0.034	0.694	−0.292	[−0.485, −0.097]
Abandonment/Instability	−0.019	0.803	−0.043	0.595	−0.025	[−0.201, 0.167]
Mistrust/Abuse	0.366	<0.001	0.227	0.009	−0.138	[−0.334, 0.060]
Social Isolation/Alienation	−0.336	<0.001	0.117	0.120	0.453	[0.269, 0.639]
Defectiveness/Shame	−0.267	0.007	0.299	0.002	0.567	[0.338, 0.793]
R^2^	0.122		0.266			
Impaired Autonomy/Performance						
Failure	−0.296	0.002	0.065	0.481	0.362	[0.135, 0.580]
Dependence/Incompetence	−0.035	0.774	0.192	0.066	0.227	[−0.046, 0.491]
Vulnerability to Harm/Illness	0.057	0.540	0.195	0.009	0.138	[−0.054, 0.325]
Enmeshment/Undeveloped Self	0.235	0.004	0.123	0.100	−0.112	[−0.283, 0.066]
R^2^	0.070		0.243			
Impaired Limits						
Entitlement/Grandiosity	0.437	<0.001	0.364	<0.001	−0.073	[−0.227, 0.082]
Insufficient Self-Control/Self-Discipline	−0.164	0.008	0.208	0.001	0.372	[0.212, 0.534]
R^2^	0.158		0.240			
Other-Directedness						
Subjugation	−0.288	<0.001	0.364	<0.001	0.652	[0.488, 0.812]
Self-Sacrifice	0.111	0.105	−0.111	0.095	−0.222	[−0.375, −0.068]
Approval/Recognition Seeking	0.411	<0.001	0.240	<0.001	−0.171	[−0.310, −0.026]
R^2^	0.155		0.221			
Overvigilance/Inhibition						
Emotional Inhibition	−0.191	0.010	0.223	0.002	0.414	[0.246, 0.568]
Unrelenting Standards/Hypercriticalness	0.285	<0.001	−0.014	0.828	−0.299	[−0.453, −0.131]
Negativity/Pessimism	−0.081	0.355	0.220	0.003	0.301	[0.110, 0.485]
Punitiveness	−0.053	0.570	0.145	0.072	0.198	[0.001, 0.403]
R^2^	0.074		0.234			

Note. N = 311. Standardized regression coefficients are reported. Adm. = Admiration; Riv. = Rivalry. qBH values are Benjamini–Hochberg-adjusted within the 18-schema Admiration and Rivalry families. Δβ = βRivalry − βAdmiration. Percentile confidence intervals for Δβ were obtained using 5000 bootstrap resamples and are unadjusted; bold Δβ values and confidence intervals denote coefficient differences that also survived Benjamini–Hochberg correction across the 18 difference tests.

**Table 3:** Hierarchical Regression Models Testing the Incremental Contribution of Alexithymia Components.

Outcome/Predictor	β	*p*
Narcissistic Rivalry		
Step 1: Schema block		
Entitlement/Grandiosity	0.304	<0.001
Defectiveness/Shame	0.342	<0.001
*R* ^2^	0.299	
Step 2: Schema + alexithymia		
Entitlement/Grandiosity	0.258	<0.001
Defectiveness/Shame	0.227	<0.001
DIF	0.184	0.006
DDF	−0.031	0.659
EOT	0.173	0.002
*R* ^2^	0.354	
Δ*R*^2^	0.055	
Δ*F*(3, 305)	8.65	<0.001
Narcissistic Admiration		
Step 1: Schema block		
Entitlement/Grandiosity	0.548	<0.001
Social Isolation/Alienation	−0.223	0.001
Emotional Inhibition	−0.218	0.001
*R* ^2^	0.263	
Step 2: Schema + alexithymia		
Entitlement/Grandiosity	0.529	<0.001
Social Isolation/Alienation	−0.239	<0.001
Emotional Inhibition	−0.239	0.001
DIF	0.103	0.152
DDF	−0.036	0.637
EOT	0.035	0.538
*R* ^2^	0.271	
Δ*R*^2^	0.007	
Δ*F*(3, 304)	1.00	0.395

Note. N = 311. Standardized regression coefficients are reported. DIF = Difficulty Identifying Feelings; DDF = Difficulty Describing Feelings; EOT = Externally Oriented Thinking.

**Table 4:** Final Structural Models of Narcissistic Rivalry and Admiration.

Outcome/Path	β	95% Bootstrap CI
Rivalry model		
Entitlement → DIF	0.179	[0.061, 0.290]
Defectiveness/Shame → DIF	0.371	[0.261, 0.470]
Entitlement → EOT	0.082	[−0.015, 0.183]
Defectiveness/Shame → EOT	0.345	[0.237, 0.464]
Entitlement → Rivalry	0.313	[0.194, 0.433]
Defectiveness/Shame → Rivalry	0.240	[0.089, 0.394]
DIF → Rivalry	0.191	[0.075, 0.302]
EOT → Rivalry	0.169	[0.042, 0.288]
Defectiveness → DIF → Rivalry	0.071	[0.027, 0.116]
Defectiveness → EOT → Rivalry	0.058	[0.014, 0.112]
Defectiveness total indirect	0.129	[0.068, 0.199]
R^2^ Rivalry	0.446	
Admiration model		
Entitlement → Admiration	0.605	
Social Isolation → Admiration	−0.244	
Emotional Inhibition → Admiration	−0.246	
R^2^ Admiration	0.323	

Model fit: Rivalry: χ^2^(8) = 31.40, CFI = 0.961, TLI = 0.899, RMSEA = 0.097, 90% CI [0.063, 0.134], pclose = 0.014, SRMR = 0.038. Admiration: χ^2^(6) = 15.26, CFI = 0.986, TLI = 0.965, RMSEA = 0.071, 90% CI [0.027, 0.115], pclose = 0.186, SRMR = 0.028.

## Data Availability

The data that support the findings of this study are available from the corresponding author upon reasonable request.
